# Primary Coenzyme Q10 Deficiency-7 and Pathogenic *COQ4* Variants: Clinical Presentation, Biochemical Analyses, and Treatment

**DOI:** 10.3389/fgene.2021.776807

**Published:** 2022-01-26

**Authors:** Jieqiong Xie, Jiayang Jiang, Qiwei Guo

**Affiliations:** ^1^ United Diagnostic and Research Center for Clinical Genetics, Women and Children’s Hospital, School of Medicine and School of Public Health, Xiamen University, Xiamen, China; ^2^ School of Medicine, Huaqiao University, Quanzhou, China

**Keywords:** COQ4, c.370G>A variant, phenotype-genotype correlation, primary coenzyme Q10 deficiency, exon

## Abstract

Primary Coenzyme Q10 Deficiency-7 (COQ10D7) is a rare mitochondrial disorder caused by pathogenic *COQ4* variants. In this review, we discuss the correlation of *COQ4* genotypes, particularly the East Asian-specific c.370G > A variant, with the clinical presentations and therapeutic effectiveness of coenzyme Q10 supplementation from an exon-dependent perspective. Pathogenic *COQ4* variants in exons 1–4 are associated with less life-threating presentations, late onset, responsiveness to CoQ10 therapy, and a relatively long lifespan. In contrast, pathogenic *COQ4* variants in exons 5–7 are associated with early onset, unresponsiveness to CoQ10 therapy, and early death and are more fatal. Patients with the East Asian-specific c.370G > A variant displays intermediate disease severity with multi-systemic dysfunction, which is between that of the patients with variants in exons 1–4 and 5–7. The mechanism underlying this exon-dependent genotype-phenotype correlation may be associated with the structure and function of COQ4. Sex is shown unlikely to be associated with disease severity. While point-of-care high-throughput sequencing would be useful for the rapid diagnosis of pathogenic *COQ4* variants, whereas biochemical analyses of the characteristic impairments in CoQ10 biosynthesis and mitochondrial respiratory chain activity, as well as the phenotypic rescue of the CoQ10 treatment, are necessary to confirm the pathogenicity of suspicious variants. In addition to CoQ10 derivatives, targeted drugs and gene therapy could be useful treatments for COQ10D7 depending on the in-depth functional investigations and the development of gene editing technologies. This review provides a fundamental reference for the sub-classification of COQ10D7 and aim to advance our knowledge of the pathogenesis, clinical diagnosis, and prognosis of this disease and possible interventions.

## Introduction

Coenzyme Q (CoQ) is a lipophilic molecule composed of 1, 4-benzoquinone and a tail of isoprenoid units. The length of the isoprenoid tail is species specific, and in humans, it is 10 units long (CoQ10). CoQ10 is ubiquitously distributed in all cells and is mostly located in the mitochondrial inner membrane, where it plays an important role in the mitochondrial respiratory chain (MRC). The oxidized form of CoQ10 (ubiquinone) can be reduced to ubiquinol by the addition of two electrons, which functions as an electron carrier that shuttles electrons from MRC complexes I (NADH: ubiquinone oxidoreductase) and II (succinate dehydrogenase) to complex III (decylubiquinol cytochrome c oxidoreductase) (Turunen, et al., 2004; Alcazar-Fabra, et al., 2016; Wang and Hekimi 2016). In addition, CoQ10 has been suggested to be involved in several other biological processes, such as pyrimidine biosynthesis (Evans and Guy 2004), *β*-oxidation of fatty acids (Watmough and Frerman 2010), structural stabilization of other MRC complexes (Cramer, et al., 2011; Letts and Sazanov 2017), production of reactive oxygen species (Guaras, et al., 2016), and inhibition of ferroptosis (Bersuker, et al., 2019; Doll, et al., 2019).

Although some CoQ10 can be obtained through dietary sources, due to its poor distribution and bioavailability, endogenous synthesis is the major source of CoQ10 (Stefely and Pagliarini 2017). Primary CoQ10 deficiency, which is defined as reduced levels of CoQ10 in tissues due to impairment of CoQ10 biosynthesis, results in a group of rare, clinically heterogeneous disorders presenting as multisystem manifestations (Desbats, et al., 2015; Quinzii, et al., 2014). To date, more than 10 genes (*ADCK3*, *PDSS1*, *PDSS2*, *COQ2*, *COQ3*, *COQ4*, *COQ5*, *COQ6*, *COQ7*, *COQ8A*, *COQ8B*, *COQ9*, *COQ10A*, and *COQ10B*) have been suggested to be involved in human CoQ10 biosynthesis (Shalata, et al., 2019; Brea-Calvo et al., 2021). Theoretically, pathogenic variants in any of these genes would result in primary CoQ10 deficiency. Based on the predicted prevalence of CoQ10 deficiency derived from the allelic frequencies of pathogenic variants, there are >120,000 individuals with CoQ10 deficiency, and most of these are undiagnosed (Hughes, et al., 2017). The clinical profile of primary CoQ10 deficiency is very complex, as there are not only pathogenic variants in different causative genes but also different pathogenic variants in the same causative gene, which result in highly heterogeneous manifestations with different ages of onset and outcomes (Alcazar-Fabra, et al., 2021). An exhaustive profile of primary CoQ10 deficiency has been recently reviewed elsewhere (Alcazar-Fabra, et al., 2021).

Among the causative genes of primary CoQ10 deficiency, *COQ4* (MIM 612898) is relatively newly confirmed, and its exact function remains largely unknown. *COQ4* is located on chromosome 9q34.11 and encodes a ubiquitously expressed 265-amino acid COQ4 protein, which is mainly localized to the matrix side of the mitochondrial inner membrane (Casarin, et al., 2008). Based on the finding using a yeast model, it has been speculated that COQ4 is essential for the stabilization of a multi-heteromeric complex containing several CoQ10 biosynthetic enzymes rather than functioning as a catalytic enzyme (Marbois, et al., 2009). According to the literature, the first patient with a *COQ4* variant and primary CoQ10 deficiency was reported by Salviati et al.(2012), in 2012; they reported a boy with a severe encephalomyopathic disorder carrying a *de novo* heterozygous 3.9 Mb deletion affecting at least 80 genes, including *COQ4*. Thus, haploinsufficiency of *COQ4* was considered to be the cause of CoQ10 deficiency in this patient (Salviati, et al., 2012). In 2015, Brea-Calvo et al. (2015) and Chung et al. (2015) further validated the association between *COQ4* variants and primary CoQ10 deficiency. Their studies demonstrated that the pathogenicity of *COQ4* variants has an autosomal recessive mode of inheritance (Chung, et al., 2015; Brea-Calvo, et al., 2015). Subsequently, the primary CoQ10 deficiency caused by *COQ4* variants was termed primary CoQ10 deficiency-7 (COQ10D7). With the increasing use of clinical whole exome sequencing (WES), more COQ10D7 patients with various phenotypes and causative variants have been found worldwide (Finsterer and Zarrouk-Mahjoub 2017; Romero-Moya et al., 2017a; Romero-Moya, et al., 2017b; Sondheimer, et al., 2017; Bosch, et al., 2018; Caglayan, et al., 2019; Ge, et al., 2019; Ling, et al., 2019; Lu, et al., 2019; Yu, et al., 2019; Chen, et al., 2020; Tsang, et al., 2020; Hashemi, et al., 2021; Mero, et al., 2021). In 2019, we reported a Chinese family with a c.370G > A *COQ4* variant along with an associated Leigh syndrome phenotype, the results of biomedical analyses, and intervention (Lu, et al., 2019). The c.370G > A variant has been shown to be East Asian population-specific, with an allele frequency of 0.001504 (gnomAD database), and was validated as a founder variant in the southern Chinese population, which was inherited from a common ancestor approximately 27 generations ago (Yu, et al., 2019). At the time this review was prepared (1 June, 2021), 28 *COQ4* variants had been reported in 38 patients, and 15 of these patients (∼40%) carried the c.370G > A variant, highlighting the high prevalence of this founder variant. Although the current number of reported patients is limited, these data will enable us to take a small step towards creating a comprehensive profile for this monogenic disease. In this review, we focus on the correlation of *COQ4* genotypes, particularly the East Asian-specific c.370G > A variant, with the clinical presentations and therapeutic effectiveness of treatments from an exon-dependent perspective, and discuss the associated biochemical analyses with an aim to advance our knowledge of the pathogenesis, clinical diagnosis, prognosis, and intervention of this disease.

## Clinical Presentation of COQ10D7

**FIGURE 1:**
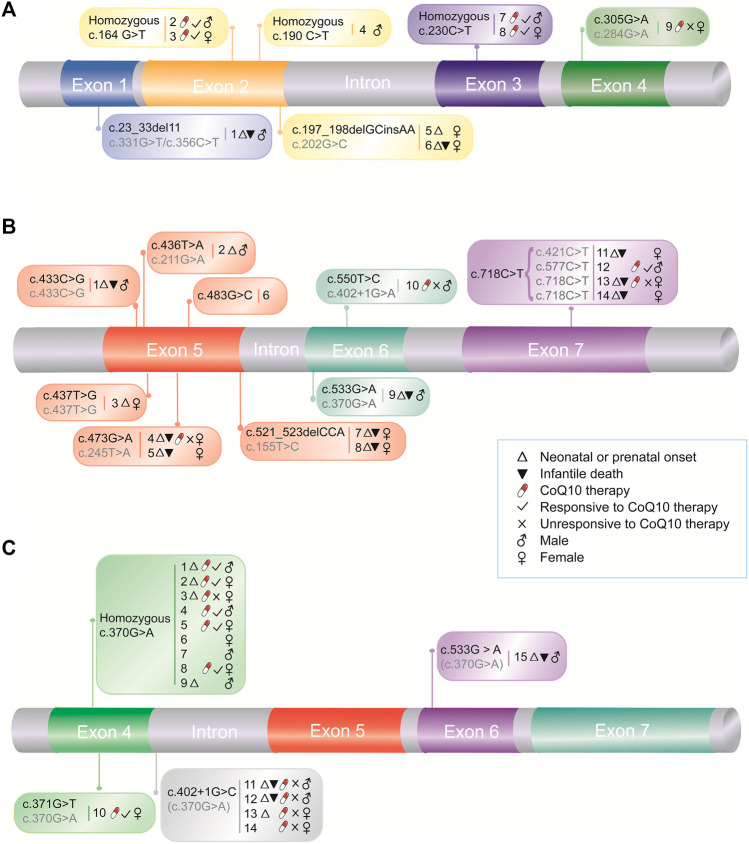
Pathogenic *COQ4* variants and associated medical information. Medical information for patients with *COQ4* variants in exons 1–4 **(A)** and exons 5–7 **(B)**, and with East Asian-specific c.370G > A variant **(C)**, respectively. The numbers indicate case numbers in Tables 1, 2, and 3. The second alleles were indicated in grey.

Similar to that of primary CoQ10 deficiency, the phenotypic spectrum of COQ10D7 is wide. We categorized the reported patients into three cohorts based on the location of their pathogenic variants: cohort 1, which includes nine patients carrying variants in exons 1–4 in both alleles except for those carrying the c.370G > A variant (Figure 1A and Table 1); cohort 2, which includes 14 patients carrying variants in exons 5–7 (Figure 1B and Table 2); and cohort 3, which consists of 15 patients carrying the c.370G > A variant (Figure 1C and Table 3). It should be noted that a compound heterozygous patient (c.370G > A/c.533G > A) was analyzed in both cohorts 2 and 3, and a patient who carried a *de novo* heterozygous complete deletion of *COQ4* was not included in any cohort (Salviati, et al., 2012).

**TABLE 1:** Clinical presentation, treatment, outcomes, and brain findings for patients with *COQ4* variants in exons 1-4.

**Case number**	1	2	3	4	5	6	7	8	9
Reference	Sondheimer et al. (2017)	Caglayan et al. (2019)	Caglayan et al. (2019)	Brea-Calvo et al. (2015)	Chung et al. (2015)	Chung et al. (2015)	Bosch et al. (2018)	Bosch et al. (2018)	Mero et al. (2021)
*COQ4* variants	c.23_33del11/c.331G>T/c.356C>T	Homozygous c.164 G>T	Homozygous c.164 G>T	Homozygous c.190C>T	c.197_198delGCinsAA/c.202G>C	c.197_198delGCinsAA/c.202G>C	Homozygous c.230C>T	Homozygous c.230C>T	c.284G>A/c.305G>A
Affected exons	1,4	2	2	2	2	2	3	3	3, 4
Demography	Unclear	Turkey	Turkey	Italian	Caucasian	Caucasian	Unclear	Unclear	Unclear
Sex	Male	Male	Female	Male	Female	Female	Male	Female	Female
**Clinical presentations**									
Onset age	At birth	8 years	8 years	10 months	At birth	Before birth	4 years	9 years	5 years
Seizures	√	√	√	√	√		√	√	√
Ataxia		√	√	√			√	√	√
Cognitive defect		√	√	√			√		√
Spasticity		√	√	√					
Oromotor dysfunction				√	√	√			
Respiratory distress	√				√	√			
Hypotonia	√				√	√			
Developmental delay					√		√		
Lactic acidosis (lactate level)	√(4-6 mM)					√(6.2 mM)			
Cardiomyopathy	√				√				
Other symptoms	Hearing loss			Scoliosis					
Brain findings	First week: focal regions of cortical increased T1 signal and MRS identified enlarged lactate peaks. Tenth week: microcephaly with volume loss and increasing prominence of lactate peaks.	Age 26 years: brain MRI showed cortical and subcortical T2 hyperintensity, not limited to a specific vascular territory.	Age 27 years: brain MRI showed cerebral and cerebellar atrophy.	Age 12 years: MRI showed bilateral increased signal intensity in FLAIR and T2W sequencing in both occipital cortical and juxtacortical areas; Electrophysiological examination showed a sensory motor polyneuropathy with slowed conduction velocities. Age 17 years: MRI showed cerebellar atrophy, widened ventricular space, scars from cortical necrotic lesions in both occipital areas.	First day: cerebellar hypoplasia, prominent extra axial space in posterior fossa and mild lateral ventricle enlargement; EEG showed multiple areas of amplitude suppression, suggesting a generalised encephalopathy and multifocal-onset seizures.	Fetal MRI: normal intracranial anatomy, transverse cerebellar diameter 10–15th percentile. Age 2 days: MRI showed decreased cerebellar hemisphere volume.	Age 5 years: brain MRI revealed a suspected tectal glioma (treated with radiotherapy)	Age 10 years: cavernoma in the left parietal lobe. Age 13 y: lesion at left occipital lobe with clear diffusion restriction.	Age 15 years: brain and spine MRI showed discrete enlargement of the pericerebellar sulci without focal abnormalities in supratentorial structures.
**Treatment**									
Symptomatic treatment	Phenobarbital, topiramate, clobazam	Carbamazepine, clonazepam	Levatiracetam					Carbamazepine	Valproate
CoQ10 supplement		2000 mg/day	2000 mg/day				1000 mg/day	1000 mg/day	ubiquinol, 100 mg/kg/day
Age at initiating CoQ10 supplement		26 years	27 years				13 years	11 years	19 years
Responsiveness to CoQ10 therapy		Marked improvement in the SARA score.	An improvement in the SARA score.				Walk test stable over the period of a year.	Walk test stable over the period of a year.	No improvement in the SARA score.
**Outcomes**	Died of acidosis and respiratory failure at the age of 4 months.	Alived at the age of 27 years.	Alived at the age of 28 years.	Alived at the age of 18 years.	Died of cardiorespiratory failure at the age of 19 months.	Died at the age of 10 weeks.	Alived at the age of 15 years.	Alived at the age of 14 years.	Alived at the age of 19 years.

SARA, the scale for the assessment and rating of ataxia; MRI, magnetic resonance imaging; MRS, magnetic resonance spectroscopy; EEG, electroencephalograph.

**TABLE 2:** Clinical presentation, treatment, outcomes, and brain findings for patients with *COQ4* variants in exons 5-7.

Case number	1	2	3	4	5	6	7	8
References	Brea-Calvo et al. (2015)	Ge et al. (2019)	Hashemi et al. (2021)	Chung et al. (2015)	Chung et al. (2015)	Romero-Moya et al. (2017a)	Brea-Calvo et al. (2015)	Brea-Calvo et al. (2015)
*COQ4* variants	Homozygous c.433C>G	c.211G>A/c.436T>A	Homozygous c.437T>G	c.245T>A/c.473G>A	c.245T>A/c.473G>A	c.483G>C	c.155T>C/c.521_523delCCA	c.155T>C/c.521_523delCCA
Affected exons	5	3, 5	5	3, 5	3, 5	5	2, 5	2, 5
Demography	Italian	Chinese	Iranian	Caucasian–Hispanic ancestry	Caucasian–Hispanic ancestry	Unclear	Austrian	Austrian
Sex	Male	Male	Female	Female	Female	Female	Female	Female
**Clinical presentations**								
Onset age	At birth	At birth	At birth	At birth	At birth	Before 4 years	Before birth	Before birth
Lactic acidosis (lactate level)	√(20.1 mM)		√	√(19.5mM)	√(22 mM)		√(6.4-14 mM)	√(3.5-9 mM)
Respiratory distress	√	√	√	√	√		√	√
Cardiomyopathy	√	√		√	√			
Hypotonia	√		√	√				
Seizures		√	√	√			√	√
Oromotor dysfunction		√	√					
IUGR								
Developmental delay		√						
Ataxia			√					
Cognitive defect			√					
Other symptoms	Areflexia, acrocyanosis, bradycardia		Visual impairment, hearing loss, sensorimotor polyneuropathy			Severe metabolic, mitochondrial defects	Distal arthrogryposis	
Brain findings		Age 3 months: head MRI showed bilateral brain atrophy, bilateral occipital parietal lobes, bilateral basal ganglia, bilateral cerebro-foot small patches DWI hyperintensity (cytotoxic edema); white matter myelin development lags behind the same age.	Age 9 years: brain MRI revealed cerebellar atrophy, and nonspecific white matter abnormal signal intensities on T2 weighted and FLAIR sequences.	At birth: brain MRI showed small cerebellar size and diffuse T2 white matter hyperintensity, and MR spectroscopy demonstrated decreased NAA and a lactate peak.			At birth: USG brain showed cerebellar hypoplasia. Autopsy: severe olivopontocerebellar and thalamic hypoplasia and scattered cavitations in the white matter.	At birth: a cranial ultrasound confirmed cerebellar hypoplasia.
**Treatment**								
Symptomatic treatment	Dobutamine infusion			Carnitine, thiamine, riboflavin, biotin, hydroxocobalamin, dopamine, milrinone, phenobarbital, fosphenytoi, levetiracetam, clobazam.	Antiepileptics			
CoQ10 supplement				Intravenous, 20 mg/kg/day.				
Age at initiating CoQ10 supplement				2 days				
Responsiveness to CoQ10 therapy				No significant improvement.				
**Outcomes**	Died 4 hours after birth.	Unknown	Alived at the age of 9 years.	Died of acidosis and respiratory failure at the age of 2 months.	Died of acidosis and respiratory failure at the age of 2 days.	Died of lethal rhabdomyolysis at the age of 4 years.	Died of acidosis and multiorgan failure at the age of 3 days.	Died of lactic acidosis at the age of 2 days.

Case number	9	10	11	12	13	14
Reference	Ling et al. (2019)	Yu et al. (2019)	Brea-Calvo et al. (2015)	Mero et al. (2021)	Chung et al. (2015)	Chung et al. (2015)
*COQ4* variants	c.370G>A/c.533G > A	c.550T>C/c.402+1G>A	c.421C>T/c.718C>T	c.577C>T/c.718C>T	Homozygous c.718C>T	Homozygous c.718C>T
Affected exons	4, 6	6, unknown	5, 7	6, 7	7	7
Demography	Chinese	Chinese	Japanese	Unclear	Ashkenazi Jewish	Ashkenazi Jewish
Sex	Male	Male	Female	Male	Female	Female
**Clinical presentations**						
Onset age	Before birth	Before 8 months	Before birth	Before 30 months	At birth	Before birth
Lactic acidosis (lactate level)	√	√(2.5-5.9 mM)	√(11.2-18.8 mM)		√(2.4 mM)	
Respiratory distress	√		√		√	√
Cardiomyopathy	√		√	√	√	√
Hypotonia		√		√	√	√
Seizures						
Oromotor dysfunction		√			√	
IUGR	√		√			√
Developmental delay		√		√		
Ataxia				√		
Cognitive defect				√		
Other symptoms		Microcephaly, dystonia, cortical visual impairment			Apnoea, bradycardia	
Brain findings		Age 21 days, 40 days, and 1 year 4 months: mild cerebral atrophy with bilateral frontal predominance.		Age 22 months and 4 years: brain MRI scans were normal.	MRS showed elevated lactate and depressed NAA peaks; EEG showed burst suppression.	An EEG was abnormal with a burst suppression pattern. Autopsy: cerebellar and brainstem hypoplasia and microdysgenesis.
**Treatment**						
Symptomatic treatment	Caffeine				Pyridoxal phosphate, folinic acid, and riboflavin	Ampicillin, gentamicin, bicarbonate, dopamine, and dobutamine
CoQ10 supplement		√		Oral, ubiquinol, 30 mg/kg/day	15 mg/kg/day divided into two doses	
Age at initiating CoQ10 supplement				3 years	1 month	
Responsiveness to CoQ10 therapy		No significant improvement.		Improvement in the neuromuscular symptoms and cardiac health.	No significant improvement.	
**Outcomes**	Died at the age of 28 days.	Alived at the age of 3 years 7 months.	Died of lactic acidosis and heart failure at the age of 2 days.	Alived at the age of 4 years.	Died at the age of 7 weeks.	Died of cardiopulmonary arrest at the age of 4 days.

IUGR, intrauterine growth retardation; DWI, diffusion-weighted imaging; FLAIR, fluid attenuated inversion recovery; MRI, magnetic resonance imaging; MRS, magnetic resonance spectroscopy; MR, magnetic resonance; EEG, electroencephalograph; NAA, N-acetylaspartic acid; USG, ultrasonography.

**TABLE 3:** Clinical presentation, treatment, outcomes, and brain findings for patients with East Asian-specific *COQ4* c.370G > A variant.

Case number	1	2	3	4	5	6	7	8
Reference	Ling et al. (2019)	Yu et al. (2019)	Yu et al. (2019)	Yu et al. (2019)	Yu et al. (2019)	Yu et al. (2019)	Lu et al. (2019)	Lu et al. (2019)
*COQ4* variants	Homozygous c.370G>A	Homozygous c.370G>A	Homozygous c.370G>A	Homozygous c.370G>A	Homozygous c.370G>A	Homozygous c.370G>A	Homozygous c.370G>A	Homozygous c.370G>A
Affected exons	4	4	4	4	4	4	4	4
Demography	Chinese	Chinese	Chinese	Chinese	Chinese	Chinese	Chinese	Chinese
Sex	Male	Female	Female	Male	Female	Female	Male	Female
**Clinical presentations**								
Onset age	1 month	At birth	At birth	2 months	2 months	4 months	2 months	2 months
Lactic acidosis (lactate level)	√(4.1 mM)	√(24 mM)	√(2.4–3.2 mM)	√	√		√ (15.1 mM)	√ (17.6 mM)
Developmental delay	√	√	√	√	√	√	√	√
Seizures	√	√		√	√	√	√	√
Respiratory distress	√	√			√		√	√
Cardiomyopathy		√			√	√	√	
Hypotonia		√			√	√		
Dystonia			√				√	√
Oromotor dysfunction			√			√	√	√
Hearing impairment	√						√	
Ophthalmic impairment			√		√			√
IUGR								
Weak responsiveness							√	√
Spasticity			√			√		
Ataxia							√	√
Brain findings	Age 4 months: MRI showed cerebral atrophy. The auditory brainstem response test showed features of conduction delay in the central auditory pathway. The nerve conduction study showed features of early demyelinating motor neuropathy. Age 2 years: MRI showed rapid progression of bifrontal cerebral atrophy, with the involvement of bifrontal cortical grey matter and white matter.	Age 7 weeks: mild cerebellar hypoplasia, mild thinning of corpus callosum.	Age 6 months: severe cerebral atrophy.	Age 32 months: moderate cerebellar atrophy without isolated vermian hypoplasia, cerebral atrophy, symmetrical loss of cerebral white matter particularly in bilateral frontal and anterior temporal regions. Corpus callosum was thinned, basal ganglia and pons unremarkable.	Age 14 months: mild thinning of corpus callosum.	Age 1 year 2 months: mild cerebellar atrophy and cerebral atrophy, white matter cystic changes with bilateral frontal and anterior temporal predominance. Corpus callosum thinning, preserved basal ganglia and brainstem.	Age 2 months: slightly widened frontal and temporal lobe. Age 5 months 18 days: bilateral, symmetrical lesions in the midbrain.	Age 1 month: normal. Age 4 months: lesions in midbrain and basal ganglia. Age 3 year 8 months: symmetrical, patchy, low density shadow in bilateral basal ganglia and diffuse brain atrophy.
**Treatment**								
Symptomatic treatment	Phenobarbital, multiple antiepileptics	Multiple anticonvulsants			Multiple anticonvulsants		Phenobarbital, levetiracetam	Thiamine, levetiracetam, phenobarbitone, carnitine
CoQ10 supplement	200 mg, 3 times a day	Intravenous	√	√	30 mg/kg/day			Oral, 50 mg/kg/day
Age at initiating CoQ10 supplement	6 years	22 days	2 years	7 years	11 months			12 months
Responsiveness to CoQ10 therapy	A subjective improvement of the patient's awareness to the surroundings was the major observation.	Cardiac function stable.	No significant improvement.	Stable condition.	Some improvement in seizure control and development.			Seizures, screaming, and respiratory distress improved. No evident improvements in nystagmus, dystonia, psychomotor development, or ambulation.
**Outcome**	Died of recurrent central apnea, aspiration pneumonia, and respiratory failure at the age of 6 years.	Alived at the age of 9 months.	Died at the age of 3 years 6 months.	Alived at the age of 8 years.	Alived at the age of 1 year 6 months.	Die of sepsis at the age of 1 year 8 months.	Died at the age of 5 months.	Alived at the age of 3 years 8 months.

Case number	9	10	11	12	13	14	15
Reference	Chen et al. (2020)	Yu et al. (2019); Ling et al. (2019)	Yu et al. (2019)	Yu et al. (2019)	Yu et al. (2019)	Yu et al. (2019)	Ling et al. (2019)
*COQ4* variants	Homozygous c.370G>A	c.370G>A/c.371G>T	c.370G>A/c.402+1G>C	c.370G>A/c.402+1G>C	c.370G>A/c.402+1G>C	c.370G>A/c.402+1G>C	c.370G>A/c.533G>A
Affected exons	4	4	4, unknown	4, unknown	4, unknown	4, unknown	4, 6
Demography	Chinese	Chinese	Chinese	Chinese	Chinese	Chinese	Chinese
Sex	Male	Female	Male	Male	Female	Female	Male
**Clinical presentations**							
Onset age	1 month	6 months	Before birth	At birth	Before birth	Before birth	Before birth
Lactic acidosis (lactate level)	√	√ (2.3 mM)	√(28.36 mM)	√ (2.6 mM)	√ (10 mM)	√	√(3.6 mM)
Developmental delay	√	√	√		√	√	
Seizures	√	√	√		√	√	
Respiratory distress			√	√	√	√	√
Cardiomyopathy			√	√	√	√	√
Hypotonia	√	√	√		√	√	√
Dystonia	√	√					
Oromotor dysfunction	√				√		
Hearing impairment	√		√				
Ophthalmic impairment	√	√	√				
IUGR			√		√	√	√
Weak responsiveness	√						
Spasticity		√					
Ataxia							
**Brain findings**	Age 3 months: MRI showed increased T1 and T2 signal in the white matter of the right parietal lobe, and displasia of white matter myelin sheath; EEG showed multifocal-onset seizures.	(Yu, et al. 2019): mild cerebral and cerebellar hypoplasia. Age 35 months:small focus of T2 and FLAIR hyperintensity at the left lentiform nucleus. Age 6 months: MRS showed raised lactate peaks at bilateral basal ganglia and frontal white matter, which normalized at the age of 7 months (Ling, et al. 2019): EEG showed potential epileptogenic discharge in the background, which was normalized after one month. MRI of the brain showed prominent subarachnoid and sulcal spaces of the cerebral hemispheres, which are consistent with microcephaly. Brain MRS revealed a lactate peak signal of uncertain significance.	Age 3 weeks: MRI showed symmetrical T1 and T2B hyperintensity with restricted diffusion at bilateral lentiform nuclei. Foci of restricted diffusion were also detected at bilateral frontal white matter. MRS showed raised lactate peaks at bilateral basal ganglia and cerebral white matter. Subsequent follow-up MRI showed established infarcts with cystic changes at bilateral lentiform nuclei. Mild cerebellar hypoplasia was also noted.		Neonatal stage: symmetrical T1 hyperintensity at bilateral basal ganglia, mild cerebellar hypoplasia, later with generalized progressive cerebellar and cerebral atrophy with diffuse white matter loss, thinning of corpus callosum. Cystic changes within cerebral white matter, bilateral basal ganglia, and thalami. MRS showed raised lactate peaks at bilateral basal ganglia. Age 9 months: MRI showed generalized progressive cerebellar and cerebral atrophy, with diffuse white matter loss including thinning of the corpus callosum. Cystic changes were seen within the cerebral white matter, bilateral basal ganglia, and thalami.		
**Treatment**							
Symptomatic treatment	Phenobarbital, levetiracetam	Steroids, levetiracetam	Phenobarbitone, levetiracetam	Carnitine			Caffeine
CoQ10 supplement	Oral, 30 mg/kg/day.	Ubiquinol, 250 mg/day; then increased to 400 mg/day. After a year, the regimen was switched to liquid liposomal ubiquinol at a dose of 100 mg/day.	40 mg/kg/day	15 mg/kg/day	√	√	
Age at initiating CoQ10 supplement	5 months	9 months	5 months	2 days	4 years 5 months	1 years	
Responsiveness to CoQ10 therapy	Unclear	Improvement in responsiveness andseizures.	No significant improvement.	No significant improvement.	No significant improvement.	No significant Improvement.	
**Outcome**	Alived at the age of 5 months.	Alived at the age of 3 years.	Died of respiratory distress at the age of 8 months.	Died at the age of 2.5 days.	Alived at the age of 4 years 6 months.	Died of respiratory failure at the age of 1 year 1 month.	Died at the age of 28 days.

IUGR, intrauterine growth retardation; MRI, magnetic resonance imaging; MRS, magnetic resonance spectroscopy; CT, computed tomography; EEG, electroencephalograph; IUGR, intrauterine growth retardation.

### Presentations in Patients With *COQ4* Variants in Exons 1–4 in Both Alleles

Three of the nine (33.3%) patients in cohort 1 (Table 1) had neonatal onset (onset within 1 month of age), and two (66.7%) of these had infantile death (death between 1 month and 1 year of age). One patient with neonatal onset and infantile death carried a frameshift variant (c.23_33del11) with a loss of function. In contrast, five other patients (5/9, 55.6%) had late onset (at age 4–8 years) and were alive at 14–28 years, suggesting a relatively mild manifestation of the disease. The most frequent presentations in this cohort were less life-threating and included central nervous system (CNS) symptoms, such as seizures (8/9, 88.9%), ataxia (6/9, 66.7%), and cognitive defects (5/9, 55.6%). In contrast, lactic acidosis (2/9, 22.2%), cardiomyopathy (2/9, 22.2%), respiratory distress or failure (3/9, 33.3%), and hypotonia (3/9, 33.3%) were the presentations of the patients who had early disease onset and death. Imaging indicated that cerebral or cerebellar abnormalities were common (8/9, 88.9%) in this cohort (Table 1). There was no sex bias in this cohort, as the female-to-male ratio was 5 to 4 (Table 1).

### Presentations in Patients With *COQ4* Variants in Exons 5–7

In contrast to cohort 1, 11 of the 14 (78.6%) patients in cohort 2 (Table 2) had neonatal onset, and 9/11 (81.8%) died in the first year of life, suggesting greater disease severity. The most frequent presentations in this cohort were multi-systemic, including severe lactic acidosis (10/14, 71.4%), respiratory failure or distress (11/14, 78.6%), cardiomyopathy (9/14, 64.3%), and hypotonia (7/14, 50.0%). Similar to the findings in cohort 1, these presentations were associated with early onset and death, suggesting that they are more life-threatening. CNS symptoms, such as seizures (5/14, 35.7%), ataxia (2/14, 14.3%), and cognitive defects (2/14, 14.3%) were less frequent, probably because these presentations are relatively late-onset and thus undetectable due to early death. The main imaging findings were cerebral and cerebellar abnormalities (7/9, 77.8%). In rare cases, abnormalities in the basal ganglia (1 case) and brainstem (1 case) were noted (Table 2). The female-to-male ratio in this cohort was 9 to 5, indicating a sex bias (Table 2), which suggests that male patients have greater disease severity and may have a higher prenatal lethality rate. However, seven of the nine patients with infantile death were females, suggesting that females were more sensitive to the disease (Table 2). Therefore, whether sex contributes to disease severity remains to be further investigated.

### Presentations in Patients With the East Asian-specific *COQ4* c.370G > A Variant

Nine patients in cohort 3 (Table 3) were homozygous for c.370G > A, and six patients were compound heterozygotes. Four of the nine (44.4%) homozygous patients had neonatal onset, but only 1 (25%) had infantile death. Among the five patients who were alive at the last follow-up, the oldest patient was ∼8 years old. The six compound heterozygous patients carried the c.370G > A variant with c.371G > T, c.402+1G > C, and c.533G > A variants. The c.371G > T variant resulted in a different amino acid change (p.Gly124Val) at the same codon as the c.370G > A variant (p.Gly124Ser). The patient with this genotype had neonatal onset and was alive at 3 years of age, which was the last follow-up. Similar to the patient with a loss of function frameshift variant (c.23_33del11) in cohort 1, three of four (75%) patients with the splicing variant c.402+1G > C had neonatal onset, and two (66.7%) of them had infantile death, suggesting that this splicing variant could be a loss of function variant, which is associated with greater disease severity. As was observed in cohort 2, the patient with the compound heterozygous c.370G > A/c.533G > A variants had neonatal onset and infantile death. The most prevalent presentations among the c.370G > A homozygotes and heterozygotes were similar and included multi-systemic symptoms, such as lactic acidosis (14/15, 93.3%), developmental delay (13/15, 86.9%), seizures (12/15, 80.0%), respiratory distress (10/15, 66.7%), cardiomyopathy (9/15, 60.0%), hypotonia (9/15, 60.0%), and dystonia (5/15, 33.3%). Similar to the variation in clinical presentations, imaging showed more diverse abnormalities in this cohort than in cohorts 1 and 2 (Table 3). The frequently affected tissues included the cerebrum (7/12, 58.3%), cerebellum (6/12, 50.0%), and corpus callosum (5/12, 41.7%). Noticeably, abnormalities in the basal ganglia and midbrain were also noted in five (5/12, 41.7%) patients, including two c.370G > A homozygotes and three c.370G > A heterozygotes, suggesting that a specific Leigh syndrome-like phenotype is correlated with this genotype (Table 3). Therefore, it can be concluded that the East Asian-specific c.370G > A variant conferred intermediate disease severity that was between the severity of the patients with variants in exons 1–4 and 5–7. Lastly, similar to cohort 1, the female-to-male ratios for c.370G > A homozygous and heterozygous patients were 5 to 4 and 3 to 3, respectively, indicating no sex bias.

## Biochemical Analyses of Patients With COQ10D7

Elevated lactate levels are frequently detected in the plasma or serum of patients with primary CoQ10 deficiency, including COQ10D7, indicating metabolic impairment. As shown in Tables 1, 2, and 3, elevated levels of lactate were observed in the three cohorts. However, greater elevation of lactate was observed in cohorts 2 and 3, in accordance with the frequent manifestation of lactic acidosis, early onset, and worse outcomes among patients in cohorts 2 and 3. In contrast, data on plasma or serum CoQ10 levels are limited. However, data from three patients indicated relatively low CoQ10 levels in serum samples (Caglayan, et al., 2019; Yu, et al., 2019).

In addition to plasma and serum biomarkers, MRC activity was evaluated in several patients and was considered to be a diagnostic indicator of primary CoQ10 deficiency. Generally, biochemical analysis of MRC activity is performed using a muscle biopsy or cultured skin fibroblasts. Currently, the correlations between this biochemical analysis and most *COQ4* genotypes are unknown because data on the MRC activity of specific genotypes are not only limited but also derived from different sample types. However, the MRC activity profiles of patients with COQ10D7 are traceable according to sample type. Generally, reduced CoQ10 levels were observed in both muscle biopsies and fibroblasts from patients with COQ10D7. MRC complex activity testing results derived from fibroblasts were highly consistent among patients, and complex II + III activity was characteristically decreased (Table 4). These results were reasonable because the shuttling of electrons from complexes I and II to complex III was most likely affected by CoQ10 deficiency. Complex I + III activity was not evaluated in most cultured cells, as the results would be unreliable (Spinazzi, et al., 2012). In contrast, MRC complex activity levels in muscle biopsies varied among patients, although reduced complex II + III activity was also common (Table 5). Interestingly, elevated levels of complexes I, III, and IV were reported in frozen post-mortem muscle samples. These results indicate that reduced levels of CoQ10 and complex II + III activity in muscle or fibroblast samples are hallmarks of COQ10D7, and these biochemical changes were more specific in fibroblasts than in muscle samples, which is consistent with the conclusion of a previous study (Montero, et al., 2008).

**TABLE 4:** Biochemical analysis of skin fibroblasts.

Case number	1	2	3	4	5	6	7	8
Reference	Sondheimer, et al. (2017)	Bosch, et al. (2018)	Chung, et al. (2015)	Chung, et al. (2015)	Lu, et al. (2019)	Yu, et al. (2019); Ling, et al., (2019)	Chung, et al. (2015)	Chung, et al. (2015)
*COQ4* variants	c.23_33del11/c.331G > T/c.356C > T	Homozygous c.230C > T	Homozygous c.370G > A	Homozygous c.370G > A	Homozygous c.370G > A	c.370G > A/c.371G > T	c.370G > A/c.402+1G > C	c.550 T > C/c.402+1G > A
Affected exons	1, 4	3	4	4	4	4	4, unknown	6, unknown
CoQ10 level	↓ 19.1 μg/mg protein (45.4–65.7)	↓ 0.26 nmol/U (0.77–1.61)	↓ 0.4 pmol/U COX (1.64–3.32)	↓ 16.4 ng/mg protein (46.1 ± 3)	↓	↓ 0.29 nmol/U COX (1.64–3.32)	↓ 0.4 pmol/U COX (1.64–3.32)	↓ 0.63 pmol/U CS (1.04–2.92)
CI	↓ 0.63 (0.86–1.70) (nmol/min/mg protein)				N	N	N	
CI + III	↓ 0.11 (0.55–1.30) (nmol/min/mg protein)			↓ 64% of CS				
CII	N		N	↓ 90% of CS	N	N	N	N
CII + III	↓ 0.08 (0.20–0.79) (nmol/min/mg protein)		↓ 183 mU/U COX (269–781)	↓ 55% of CS	↓	↓ 135 mU/U COX (269–781)	↓ 130 mU/U COX (269–781)	↓ 183 mU/U COX (269–781)
CIII			N			N	N	N
CIV	N			↓ 67% of CS	N	N	N	
CV	N							

CI, complex I; CII, complex II; CIII, complex III; CIV, complex IV; CS, citrate synthase; COX, cyclooxygenase; N, normal; ↓ decreased level.

**TABLE 5:** Biochemical analysis of muscle biopsies.

Case number	1	2	3	4	5	6	7	8	9
Reference	Sondheimer, et al. (2017)	Brea-Calvo, et al. (2015)	Brea-Calvo, et al. (2015)	Brea-Calvo, et al. (2015)	Chung, et al. (2015)	Yu, et al. (2019); Ling, et al. (2019)	Brea-Calvo, et al. (2015)	Brea-Calvo, et al. (2015)	Chung, et al. (2015)
*COQ4* variants	c.23_33del11/c.331G > T/c.356C > T	c.155 T > C/c.521_523delCCA	c.155 T > C/c.521_523delCCA	Homozygous c.190C > T	c.245 T > A/c.473G > A	c.370G > A/c.371G > T	c.421C > T/c.718C > T	Homozygous c.433C > G	Homozygous c.718C > T
Affected exons	1, 4	2, 5	2, 5	2	3, 5	4	5, 7	5	7
CoQ10 level	↓ 6.9 μg/g tissue (19.6–46.8)				↓ 5.2 μg/g (16% of N)	N			
CI		N	↑ 145% of CS	↓ <5% of CS		N	↓ 6% of CS	↓ 36% of CS	N
CI + III		N	N					↓ 24% of CS	
CII		N	N	N		N	↓ 42% of CS	N	N
CII + III		↓ 55% of CS	N	↓ 30% of CS	↓ 14% of N		↓ 43% of CS	↓ 34% of CS	
CIII		N	↑ 222% of CS	↓ 50% of CS		N	↓ 10% of CS	N	N
CIV		↓ 50% of CS	↑ 189% of CS	N		N	↓ 30% of CS	N	N

CI, complex I; CII, complex II; CIII, complex III; CIV, complex IV; CS, citrate synthase; N, normal; ↓, decreased level; ↑, increased level.

## Treatment of COQ10D7

Early oral supplementation with high-dose CoQ10 has been reported to improve a wide spectrum of phenotypes of primary CoQ10 deficiency (Montini, et al., 2008; Hargreaves 2014). Therefore, some patients with COQ10D7 were treated with CoQ10 therapy, in the form of ubiquinone or ubiquinol, and various responses were reported.

### Treatment of Patients With *COQ4* Variants in Exons 1–4 in Both Alleles

In this cohort (Figure 1A), five patients initiated CoQ10 therapy at different ages and at different doses (1,000–∼5,000 mg/day), and four (80%) showed positive effects, including improvements in neurological condition and walking ability (Table 1). Compared to the responsive patients, the unresponsive patient carried variants in exon 4 and had a relatively early onset. As was observed in patients with the c.370G > A variant, the disease manifestations associated with changes in this exon may be more severe than those associated with variants in the upstream exons. Moreover, CoQ10 therapy was initiated relatively late (at 19 years of age). These could be the reasons for this patient’s unresponsiveness to CoQ10 therapy. Nevertheless, CoQ10 therapy was effective in most patients in this cohort.

### Treatment of Patients With *COQ4* Variants in Exons 5–7

In contrast to cohort 1, four patients in cohort 2 (Figure 1B) received CoQ10 therapy, and three (75%) were unresponsive, which is in accordance with the life-threatening presentations of patients in this cohort (Table 2). The responsive male patient had both a relatively late onset and early initiation of CoQ10 therapy, suggesting that disease severity and initiation of therapy are the essential determinants of CoQ10 therapy effectiveness.

### Treatment of Patients With the East Asian-specific c.370G > A *COQ4* Variant

Six homozygous patients in this cohort were treated with CoQ10 supplementation, and five (83.3%) (three females and two males) were responsive to the therapy (Figure 1C), suggesting that CoQ10 therapy effectively improved the health of these homozygous patients.

For the compound heterozygous patients with the c.370G > A variant, responsiveness to CoQ10 therapy depended on the other variant they carried. For example, the patient with the c.371G > T variant responded to CoQ10 therapy, which is in accordance with the similar clinical presentations of this patient and those of c.370G > A homozygotes. In contrast, none of the four patients carrying the c.402+1G > C variant benefited from CoQ10 therapy, which is also in accordance with the severe manifestations caused by this loss-of-function variant. These results further support the hypothesis that the effectiveness of CoQ10 therapy is associated with disease severity and the timing of therapy initiation.

## Discussion

The highly heterogeneous clinical profiles of primary CoQ10 deficiencies like COQ10D7, including variations in clinical manifestations, age of onset, treatment effectiveness, and outcomes, could be attributed to many speculative factors (Alcazar-Fabra, et al., 2021). For example, genetic factors, such as differences in pathogenic variations within the diverse genetic backgrounds of populations or ethnicities, epigenetic modifications, and defects in biological processes other than MRC activity as well as environmental factors, including the nutritional or metabolic status of specific tissues have been suggested. Moreover, the combined effects of these factors may manifest very early, even in the first stages of embryonic development and may eventually determine the degree of tissue damage and disease severity (Alcazar-Fabra, et al., 2021; Fabozzi, et al., 2021). Due to the limited number of cases and the evident bias in many studies, it is impossible to fully decipher the correlations between the clinical profiles and putative causes, which hampers the diagnosis and management of this set of diseases (Alcazar-Fabra, et al., 2021). However, the fragmented pieces of information derived from existing COQ10D7 cases enable us to take a small step towards creating a comprehensive profile for this monogenic disease.

### Genotype-Phenotype Correlations

Generally, the major clinical presentations caused by pathogenic *COQ4* variants in exons 1–4 (i.e., amino acid changes in the N-terminus of COQ4) are CNS symptoms which are less life-threating, and are associated with late onset, responsiveness to CoQ10 therapy, and a relatively long lifespan. In contrast, the clinical presentations caused by pathogenic *COQ4* variants in exons 5–7 (i.e., amino acid changes in the C-terminus of COQ4) included multi-systemic symptoms which are more fatal and are associated with early onset, unresponsiveness to CoQ10 therapy, and early death. Patients with the East Asian-specific c.370G > A variant displayed intermediate disease severity with multi-systemic dysfunction, which was between that of the patients with variants in exons 1–4 and 5–7. Specifically, the c.370G > A homozygous patients had early disease onset, were responsive to CoQ10 therapy, and a relatively long lifespan. For the c.370G > A heterozygotes, case with other allelic variant located in exon 4 had late onset, was responsive to CoQ10 therapy, and relatively long lifespan, whereas case with allelic variant located in exon 6 had early onset, was unresponsive to CoQ10 therapy, and early death. Moreover, loss-of-function variants, such as frameshift and splicing variants, were associated with early onset, unresponsiveness to CoQ10 therapy, and poor outcomes. Lastly, based on the current data, sex is unlikely to be associated with disease severity.

**FIGURE 2:**
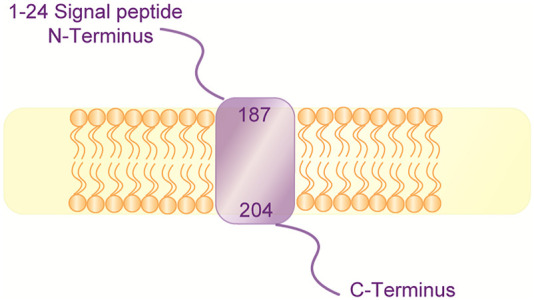
Predictive structure of COQ4.

The mechanism underlying the exon-dependent genotype-phenotype correlation may be associated with the structure and function of COQ4. Yeast Coq4, which is a functional ortholog of human COQ4, is located at the inner mitochondrial membrane on the matrix side and interacts with other Coq proteins, such as Coq3, Coq5, Coq6, Coq7, and Coq9 (Belogrudov, et al., 2001; Marbois, et al., 2005; Awad, et al., 2018). Two functional motifs have been identified in the C-terminus of Coq4; one is a geranylgeranyl monophosphate lipid-bound long hydrophobic α-helix (PDB: 3KB4, Northeastern structural genomics program), and the other is a highly conserved putative zinc ligand motif [HDxxH-(x)11-E] (Marbois et al., 2009). Coq4 is thought to function as a scaffold or organizer that simultaneously anchors the inner mitochondrial membrane, CoQ synthetic complex, and long polyisoprenyl tail of CoQ intermediates and/or CoQ, to organize the ring modifications in the CoQ biosynthetic pathways, and the C-terminus of Coq4 is essential for maintaining these functions (Marbois et al., 2009; Rea et al., 2010; He et al., 2014). In the predicted structure of human COQ4 with Phyre2 (Kelley, et al., 2015), there are transmembrane helices in the C-terminus (Figure 2). Therefore, it is reasonable to speculate that the C-terminus of human COQ4 is likely also important for protein function; thus, *COQ4* variants in exons 5–7, which are more likely to disrupt the function of the C-terminus, could cause more serious manifestations than variants in exons 1–4. Similar associations between pathogenic variants in specific domains and certain phenotypes have been also reported in other *COQ* genes. For example, missense variants within the COQ8 protein-specific KxGQ domain were shown to be associated with cortical and pyramidal tract dysfunction (Traschutz, et al., 2020). Nonetheless, more data, including a high-resolution protein structure and in-depth mechanistic investigations, are required to confirm this speculation.

Notably, a dominant mode of inheritance was suggested for two patients: a 3-year-old boy who carried a *de novo* heterozygous 3.9-Mb deletion affecting at least 80 genes, including *COQ4*, who displayed intellectual disability, encephalomyopathy, and dysmorphic features (Salviati, et al., 2012), and a 4-year-old girl with a heterozygous *COQ4* c.483 G > C variant who displayed severe metabolic/mitochondrial deficits, extensive muscle damage, and lethal rhabdomyolysis (Romero-Moya, et al., 2017b). However, some speculative reasons may explain these exceptions. For the first patient, the presence of a second allelic variant, for example, a variant in a regulatory region, would have been missed by only sequencing the exon regions. Moreover, the clinical presentations could be attributed to, either fully or in part, other causative genes other than *COQ4*, since at least 80 genes were affected by the deletion. Similarly, the presence of a second allelic variant may have been missed in the second patient, which could explain why genetic rescue of mitochondrial and skeletal muscle impairment was observed with patient-origin induced pluripotent stem cells (iPSCs) when the c.483 G > C variant was corrected. However, as mentioned above, the phenotypic heterogeneity is high among COQ10D7 patients, even for siblings who share similar genetic backgrounds, suggesting that other genetic or environmental contributors are involved in the pathogenesis of COQ10D7. Moreover, CoQ10 is involved in several biological processes, but it remains unclear whether any of the clinical presentations are attributed to the impairment of other biological processes instead of MRC deficiency in COQ10D7. Therefore, it is possible that, in rare cases, clinical manifestations could be associated with hemizygous or heterozygous *COQ4* variants.

### Biochemical Analyses

In accordance with the role of CoQ10 in the MRC, reduced levels of CoQ10 and complex II + III activity in muscle or fibroblast samples are hallmarks of COQ10D7. *In vivo* assessments of the CoQ biosynthetic rate using cultured fibroblasts were available, which enabled us to discriminate primary deficiency from secondary deficiency (Rodriguez-Aguilera, et al., 2017). Although the detection of CoQ10 biosynthesis and MRC activity have some diagnostic value, their clinical utility for COQ10D7 diagnosis is limited because of the invasive sampling, relatively long turnaround time for cell culture, limited data regarding diagnostic accuracy, and the lack of a consensus or comprehensive evaluation of detection methodologies. Therefore, genetic testing, particularly high-throughput sequencing, either whole exome and genome-based, should be used as the first-line tool to diagnose COQ10D7 in a rapid and definitive manner, especially when point-of-care high-throughput sequencing is available (Sun, et al., 2020; Sanford, et al., 2019). In fact, all reported *COQ4* variants were diagnosed using WES. However, it should be noted that, for new potentially pathogenic variants identified by genetic testing, evaluations of CoQ10 biosynthesis and MRC activity are necessary to confirm their pathogenicity (Brea-Calvo et al., 2021). Phenotypic rescue by CoQ10 treatment would also provide convincing additional evidence for the pathogenicity of a *COQ* gene variant (Romero-Moya et al., 2017a).

In addition to muscle and skin biopsies, measurements of biomarkers in plasma, serum, white blood cells, or urine show promise for clinical applications due to their less invasive sampling. Although an elevated lactate level is not specific for the diagnosis of COQ10D7, it is valuable for evaluating disease severity and prognosis. Another biomarker, serum CoQ10, was detected at relatively low levels in three COQ10D7 patients (Yu, et al., 2019; Caglayan, et al., 2019), suggesting that it could be used for the preliminary diagnosis of COQ10D7 when accompanied with clinical presentations. Moreover, assessment of plasma or serum CoQ10 is simple and rapid, and could provide the first clue and promote timely CoQ10 supplementation. However, more plasma and serum CoQ10 data are needed to establish reference intervals and to validate this concept. Although decreased plasma or serum CoQ10 is not COQ10D7 specific and is associated with all types of primary CoQ deficiency, patients with all types of primary CoQ deficiency could likely benefit from CoQ10 supplementation, and no detrimental side effects have been associated with this biomolecule (Hargreaves 2014). Nevertheless, if skin fibroblasts could be obtained less invasively, they would be an ideal option due to the analytical accuracy of their biochemical analysis and the ability of functional studies (Romero-Moya, et al., 2017b; Alcazar-Fabra, et al., 2021).

### Treatment of COQ10D7

Clear evidence supporting the use of any specific drug for the treatment of mitochondrial diseases (MD) is lacking. However, based on numerous cases with positive responses and the lack of noticeable toxicity, exogenous CoQ10 is widely used for the treatment of MD, particularly primary CoQ10 deficiency (Hargreaves 2014; Orsucci, et al., 2019). Recently, ubiquinol was approved as an orphan drug for the treatment of primary CoQ10 deficiency (Hernandez-Camacho, et al., 2018). Although the delivery and utilization of exogenous CoQ10 is inefficient because of its high molecular weight and low aqueous solubility, when CoQ10 is administered at high doses or in specific formulations, CoQ10 levels are increased in all tissues, including the heart and brain (Zaki 2016). However, approximately 43% (9/21) of patients with COQ10D7 did not benefit from CoQ10 therapy. Due to inconsistent treatment regimens, including variations in formulation and dose, and the lack of data regarding *in vivo* CoQ10 levels, it is currently impossible to determine the conclusive reasons for unresponsiveness in these patients. However, according to the limited data available, the responsiveness to CoQ10 therapy was highly associated with the location of the pathogenic *COQ4* variants and the severity of clinical presentation. A rational speculation was that CoQ10 is more likely to prevent further harm rather than reverse the present impairments. Therefore, unresponsiveness to CoQ10 therapy was more likely to be observed in patients with early onset and life-threatening manifestations. In general, CoQ10 therapy, when administered in time, would be effective, to various extents, for those patients with *COQ4* variants in exons 1–4 in both alleles, including the c.370G > A homozygotes, thus highlighting the importance of early genetic diagnosis and intervention.

For responsive patients, the symptoms of multisystem dysfunction, including the CNS, were improved, although it is difficult for CoQ10 to penetrate the blood-brain barrier (Musumeci, et al., 2001; Lamperti, et al., 2003; Artuch, et al., 2006; Mancuso, et al., 2012; Mortensen, et al., 2014). It has been shown that ubiquinol has greater therapeutic effectiveness than ubiquinone due to the improved mitochondrial bioenergetics in the brain and reduced oxidative stress and astrogliosis (Garcia-Corzo, et al., 2014). However, the differences in the therapeutic efficacy of ubiquinol and ubiquinone for COQ10D7 treatment are unclear due to limited data. Since CoQ10 is a lipophilic molecule, its gastrointestinal uptake and assimilation may be inefficient (Masotta, et al., 2019). Therefore, CoQ10 derivatives of a more absorbable nature could be promising alternatives to enhance CoQ10 biosynthesis or bypass deficient steps in the CoQ10 biosynthetic pathway (Awad, et al., 2018). Recently, *β*-resorcylic acid, a structural analog of the CoQ precursor 4-hydroxybenzoic acid (4-HB), was shown to be a powerful therapy for patients with COQ9 or COQ7 variants, thus shedding light on the application of 4-HB analogs for other forms of primary CoQ10 deficiency, including COQ10D7 (Hidalgo-Gutierrez, et al., 2019). In addition to CoQ therapy, symptomatic treatments were also helpful to some extent for improving the health condition of patients with MD. In general, there is no substantial difference in symptomatic treatments for the same conditions between patients with MD and non-MD patients (Orsucci, et al., 2019). However, in terms of COQ10D7 treatment, the effectiveness of drugs, such as creatine, thiamine, riboflavin, and l-carnitine, requires further evaluation, although some of these drugs may be associated with positive outcomes in rare cases. Moreover, other drugs, such as valproate, linezolid, and aminoglycosides, should be used carefully because of their potential for mitochondrial toxicity (Mancuso, et al., 2012; Orsucci, et al., 2019). Notably, two c.370G > A homozygous patients died due to infections and associated complications (Ling, et al., 2019; Yu, et al., 2019), suggesting that infection management is crucial for COQ10D7 patients since they could lead to metabolic crises in patients with MD (Orsucci, et al., 2019).

Based on findings regarding genotype-phenotype correlations, in-depth functional investigations of COQ4 domains would be useful for the development of targeted drugs for COQ10D7 patients with specific variants. Similarly, targeted drugs could be developed based on an understanding of the presentations associated with impairment of other biological processes besides MRC. Revolutionary advances in multifaceted omics technologies, including genomics, metabolomics, proteomics, epigenomics, transcriptomics, and interactomics, will continue to expand our knowledge of mitochondrial pathology (Rahman and Rahman 2018) and provide us with the possibility of developing targeted drugs based on newly identified molecular phenotypes.

In addition to conventional therapies, targeted gene therapy is a novel and attractive prospect for COQ10D7. With the development of induced pluripotent stem cells (iPSCs) and gene editing technologies, *in vitro* and *in vivo* disease models can be established more readily to advance the development of gene therapy (Karpe, et al., 2021). For example, phenotypic rescue was achieved in patient-derived iPSCs and mouse models via rectification of pathogenic variants in amyotrophic lateral sclerosis and disruption of splicing regulatory elements in spinal muscular atrophy (SMA) using gene editing (Wang, et al., 2017; Son, et al., 2019; Lin, et al., 2020). A similar investigation was initiated for COQ10D7 in which genetic rescue of mitochondrial and skeletal muscle impairment was attained in iPSCs with a *COQ4* c.483G > C variant (Romero-Moya et al., 2017a). Currently, three targeted drugs (nusinersen, onasemnogene abeparvovec-xioi, and risdiplam) based on gene replacement or splicing modification have been approved by the US Food and Drug Administration for use in the clinical treatment of SMA (Menduti, et al., 2020), confirming the promising clinical utility of targeted gene therapy. However, more comprehensive *in vivo* evaluations of the efficacy and safety of gene therapy for COQ10D7 are required because the genetic abnormalities in COQ10D7 are more heterozygous than those in SMA.

Whether conventional or targeted gene therapies are administered, a unified, sensitive, and valid design for measuring outcomes is a prerequisite for accurate evaluation of therapeutic efficacy (Mancuso, et al., 2017; Steele, et al., 2017; Koene, et al., 2018). Such comprehensive evaluations were lacking in previous COQ10D7 studies, partially due to the rapid deterioration of patients who have the disease. However, this should be emphasized in future studies, if possible. Randomized, double-blind, placebo-controlled, crossover trials are also essential for accurate evaluation of therapeutic efficacy in a subset of COQ10D7 patients, for example, patients who have *COQ4* variants in exons 1–4 in both alleles and present with mild symptoms.

## Conclusion and Perspectives

We showed that COQ10D7 displays distinct clinical presentations, and responsiveness to CoQ10 therapy depends on the location of *COQ4* variants, providing a fundamental reference for the sub-classification of and prognosis prediction for this disease. Moreover, sex is unlikely to be associated with disease severity. Point-of-care high-throughput sequencing would be useful for the rapid diagnosis of pathogenic *COQ4* variants, and biochemical analyses to detect the characteristic impairments in CoQ10 biosynthesis and MRC activity and, phenotypic rescue by CoQ10 treatment, are necessary to confirm the pathogenicity of suspicious variants. In addition to CoQ10 derivatives, targeted drugs and gene therapy could be useful for COQ10D7 treatment relying on in-depth functional investigations and the development of gene editing technologies.
