# Supplementary material for: Primary Coenzyme Q10 Deficiency-7 and Pathogenic COQ4 Variants: Clinical Presentation, Biochemical Analyses, and Treatment
Source: Front Genet. 2022 Jan 26;12:776807. doi: 10.3389/fgene.2021.776807 (PMC8826242; doi:10.3389/fgene.2021.776807)
Supplement: Supplementary file 1 [file DataSheet1.PDF]

·临床研究与实践·

原发性辅酶Q10缺乏7型一例并文献复习

陈先睿　许锦平　姚拥华
厦门大学附属第一医院儿科 厦门市儿科重点实验室 厦门大学医学院儿童医学研究所 361003
通信作者：姚拥华，Email：1684907959@qq.com

【摘要】 **目的**　探讨辅酶Q4（COQ4）基因变异致原发性辅酶Q10缺乏7型（COQ10D7）患儿的临床特点及基因变异情况。**方法**　对厦门大学附属第一医院儿科2020年3月1例COQ4基因变异致COQ10D7患儿的临床资料及基因结果进行分析。以"原发性辅酶Q10缺乏症""COQ4基因"和"primary coenzyme Q10 deficiency""COQ4 gene"为关键词分别检索万方数据库、中国知网、PubMed、在线人类孟德尔遗传（OMIM）、ClinVar数据库（建库至2020年4月），总结原发性COQ10D7患儿临床特征和基因变异情况。**结果**　患儿　男，5月龄，因间断抽搐3个月就诊，确诊为"癫痫"。患儿喂养困难，生长发育迟缓，四肢肌张力低下，伴有血乳酸升高。全外显子测序显示发现了具有致病意义的COQ4基因纯合变异c.370G>A。文献检索纳入临床资料齐全的中文文献1篇，外文文献9篇，共32例。汇总分析33例（包括本例）病例资料，其中12个错义变异，2个移码变异，剪切变异、无义变异及缺失变异各1个，而c.370G>A仅在中国南方地区患儿中发现。多在新生儿期（22例）起病，新生儿呼吸窘迫或呼吸功能不全20例，癫痫发作21例，肥厚性心肌病20例，血清乳酸升高或乳酸酸中毒26例，28例颅脑影像学可见大脑或小脑发育不良、脑萎缩、基底节病变等多种病变。多数患儿预后不佳，33例患儿中死亡20例，死亡年龄4小时龄~3岁6月龄。**结论**　原发性COQ10D7主要临床表型为新生儿期呼吸窘迫或呼吸功能不全、癫痫、心肌肥厚及乳酸升高，致病原因为COQ4基因变异，c.370G>A可能是中国南方地区患儿热点变异。

【关键词】　癫痫；　辅酶Q10缺乏症；　基因，COQ4

Primary coenzyme Q10 deficiency-7: a case report and literature review

Chen Xianrui, Xu Jinping, Yao Yonghua
Department of Pediatrics, the First Affiliated Hospital of Xiamen University, Pediatric Key Laboratory of Xiamen, Institute of Pediatrics, School of Medicine, Xiamen University, Xiamen 361003, China
Corresponding author: Yao Yonghua, Email: 1684907959@qq.com

【**Abstract**】　**Objective**　To explore the clinical characteristics and gene variation of primary coenzyme Q10 deficiency-7 (COQ10D7) in children. **Methods**　Clinical data and genetic tests results of a COQ10D7 child caused by coenzyme Q4 (COQ4) gene variation at the First Affiliated Hospital of Xiamen University in March 2020 were collected and analyzed. A literature search with "primary coenzyme Q10 deficiency" or "COQ4 gene" as the keyword was conducted at Wanfang database, China national knowledge infrastructure(CNKI), PubMed, online Mendelian inheritance in man (OMIM), ClinVar database (up to April 2020), the clinical characteristics and gene variation of children with primary COQ10D7 were summarized. **Results**　A 5-month-old boy was diagnosed as "epilepsy" because of intermittent epileptic seizures in three months. He had feeding difficulties, growth retardation, hypotonia of limbs and increased lactic acid. His whole exon gene testing suggested a homozygous variation of COQ4 gene (c.370G>A). One article in Chinese and 9 articles in English were found, which made up the complete case data of 33 patients (including our case). There

DOI：10.3760/cma.j.cn112140-20200610-00601
收稿日期　2020-06-10　　**本文编辑**　李伟
引用本文：陈先睿, 许锦平, 姚拥华. 原发性辅酶Q10缺乏7型一例并文献复习[J]. 中华儿科杂志, 2020, 58(11): 928-932. DOI: 10.3760/cma.j.cn112140-20200610-00601.

were 12 missense variations, 2 frameshift variations, 1 splicing variation, 1 nonsense variation and 1 deletion variation, among these variations c. 370G>A was found only in children in southern China. The age of onset was mostly in the neonatal period (22 cases). Among all patients, 20 cases had presented neonatal respiratory distress or respiratory insufficiency, 21 cases had seizures, 20 cases had hypertrophic cardiomyopathy, and 26 cases had elevated serum lactic acid or lactic acidosis. Brain dysplasia, brain atrophy, basal ganglia and other lesions were observed on brain magnetic resonance imaging in 28 cases. Most of them had a poor prognosis with a mortality rate of 20/33. The age of death ranged from 4 hours to 42 months old. **Conclusions** The main clinical phenotypes of primary COQ10D7 are neonatal respiratory distress or respiratory insufficiency, epilepsy, myocardial hypertrophy and lactic acid elevation. Primary COQ10D7 is caused by homozygous or compound heterozygous variation in the COQ4 gene, and c.370G>A may be the hotspot variation in children in southern China.

【**Key words**】 Epilepsy; CoQ10 deficiency; Genes, CoQ4

原发性辅酶Q10缺乏症是一种由线粒体功能障碍引起的常染色体隐性遗传性疾病[1]，目前已知的共有8个分型，其中原发性辅酶Q10缺乏7型[coenzyme Q10 deficiency-7，在线人类孟德尔遗传(online Mendelian inheritance in manCOQ10D7，OMIM）616276]是由辅酶Q4(coenzyme Q4，COQ4）基因变异引起。COQ4基因是编码辅酶Q生物合成的关键因子，具有催化辅酶Q10生物合成的重要作用[2]，其变异可引起辅酶Q10缺乏，从而导致神经肌肉疾病。大多数患儿在出生后不久就出现严重的心脏或神经症状，组织样本显示辅酶Q10水平下降[1]。该病常见的临床表型包括新生儿呼吸功能不全、喂养困难、癫痫性脑病、肥厚性心肌病、左室发育不全、心动过缓、动脉导管未闭、小脑发育不全、感觉运动性多发性神经病等[3]。国内外关于COQ4基因变异导致的原发性辅酶Q10缺乏症的文献报道较少[1-2]。现报道1例原发性COQ10D7的临床特点，并结合文献复习进行分析，以提高临床医师对COQ10D7的认识和诊疗水平。

病例资料

一、本组病例资料

患儿 男，5月龄，因间断抽搐3个月于2020年3月就诊厦门大学附属第一医院儿科神经专科。患儿1月龄时出现双眼凝视，伴双侧上下肢肌肉阵发性痉挛或抖动，呈丛集性发作，10余次/d，每次持续时间1~2 h，纳差、反应差，当地医院诊断“癫痫”，予苯巴比妥止惊、抗感染等治疗后好转出院。出院后仍有间断抽搐，症状基本同前，追听、追物差，甚至不追物，对声音、疼痛刺激反应差，生长发育落后，期间再次就诊外院诊断“癫痫、发育协调障碍、视觉障碍、预激综合征”，予左乙拉西坦(0.4 ml，2次/d）口服抗癫痫，康复治疗后好转出院。期间癫痫仍有间断发作，4月龄时就诊厦门大学附属第一医院儿科门诊后予调整左乙拉西坦剂量(1.5 ml，2次/d）口服继续抗癫痫，同时完善基因检查后结果回报提示原发性COQ10D7，予大剂量辅酶Q10 100 mg/次，3次/d，30 mg/(kg·d）口服治疗。门诊体格检查：身长62 cm(低于同龄儿童2个标准差），体重5.85 kg(低于同龄儿童2个标准差），头围37.5 cm。无特殊外貌，精神一般，心、肺、腹无异常，肌张力减低，病理征阴性。既往史：出生后10 h出现喂养困难外院新生儿科住院，诊断为“新生儿惊厥、新生儿高胆红素血症”，予苯巴比妥止惊、光疗降低血清未结合胆红素4 d后，黄疸消退、未再惊厥，予出院。患儿为其母第6胎第3产，足月自然出生，出生体重3.25 kg，无窒息抢救史。出生后混合喂养。患儿次兄3月龄因“发育障碍并惊厥”就诊后确诊癫痫，予左乙拉西坦治疗，6岁余死亡。长兄10岁，健康。其母亲的另外3次怀孕均自愿终止妊娠。父母体健，否认近亲婚配。

辅助检查：心脏彩色超声(患儿3月龄)示房间隔卵圆孔未闭。头颅磁共振成像(magnetic resonance imaging，MRI)示右顶叶白质内片状长T1长T2信号、白质髓鞘发育落后。血乳酸2.0 mmol/L(参考值1.33~1.78 mmol/L)。血氨77.5 μmol/L(参考值10~47 μmol/L)。血氨基酸未见异常。尿有机酸中草酸-2，磷酸-3.3-甲基戊烯二酸-2.2-羟基戊二酸-3，2-羟基已二酸-3升高。脑电图示异常婴儿脑电图，清醒期双侧枕区，左侧颞区、顶区、颞区散发多量尖波，尖慢综合波。

征得父母同意并签署知情同意书。抽取患儿及其父母外周血标本，送检北京赛福解码基因科技

有限公司基于NovaSeq 6000技术测序平台，采用IDT xGen Exome Research Panel进行捕获建库，双末端测序策略。致病变异位点筛选原则：(1)筛选出外显子区变异、非同义变异位点。(2)ExAC_EAS、ExAC_ALL、千人基因组、人群频率数据库(genome aggregation database，gnomAD)等数据库中未见正常人携带或携带率小于5%。(3)参考dbSNP、OMIM、人类基因突变数据库(human gene variant database，HGMD)、ClinVar等多种数据库对致病变异位点进行评估。(4)使用SIFT、Polyphen2、LRT、MutationTaster、FATHMM等多种蛋白功能预测软件进行基因变异导致蛋白功能预测。根据美国医学遗传学与基因组学学会(American College of Medical Genetics and Genomics，ACMG)分类指南以及患儿的临床表型进行致病变异的筛选[4]。使用二代测序技术检出的与患儿临床表型相关的致病变异位点会采用一代Sanger测序技术进行验证，同时进行双亲样本验证。患儿发现了具有致病意义的COQ4基因第9外显子内的纯合变异c.370G>A，分别来源于其父亲和母亲，见图1。利用Polyphen-2蛋白功能预测软件预测该变异位点，预测值为0.99和0.90，预测结果很可能有害(该变异对蛋白影响有害)，同时PROVEAN软件预测结果也提示有害。

二、文献复习

以“原发性辅酶Q10缺乏症”“COQ4基因”和“primary coenzyme Q10 deficiency”或“COQ4 gene”为关键词检索万方数据库、中国知网、PubMed、OMIM、ClinVar数据库(建库至2020年5月)，检索到中文文献1篇[2]，英文文献9篇[1, 3, 5-11]，共35例COQ4基因变异致病患儿。其中8篇文献报道了32例患儿的详细临床资料[1-3,5-9]，11例患儿来自中国台湾或香港[1]，3例来自中国大陆地区[2-3]，余18例为国外报道[5-9]。现有研究报道原发性COQ10D7相关的COQ4基因变异仅有17个(HGMD，http://www.hgmd.cf.ac.uk/ac/index.php)，变异类型中12个错义变异，2个移码变异，1个剪切变异，1个无义变异，1个缺失变异。

总结33例(包括本例)COQ4基因变异致病的原发性辅酶Q10缺乏症患儿临床特点，男12例、女21例，多在新生儿期(22例)或婴儿期(9例)起病。22例新生儿期起病患儿中有20例(91%)新生儿呼吸窘迫或呼吸功能不全且和早产无关；12例(36%)患儿出现不同程度的喂养或吞咽困难；17例(52%)患儿具有精神或生长发育障碍，部分以语言发育落

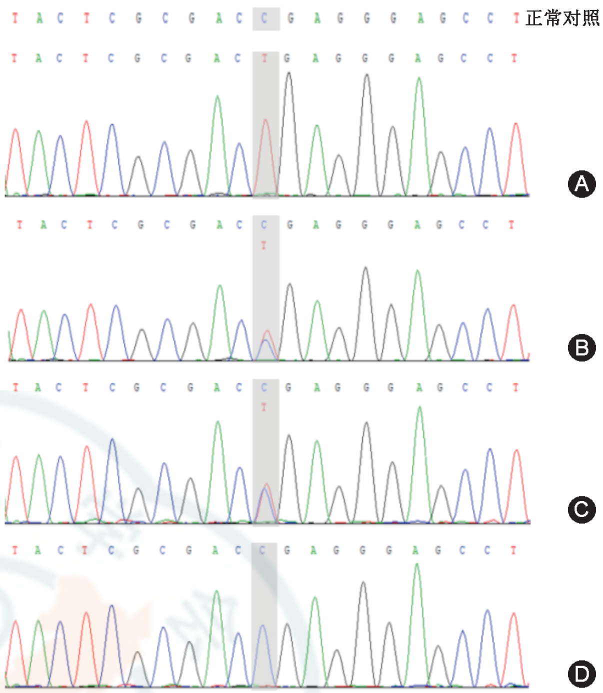

注：由于Sanger验证采用反向测序，峰图显示的碱基为被检测碱基的反向互补序列；阴影示变异位点

图1　原发性辅酶Q10缺乏7型患儿及其家系的基因测序图　A：患儿辅酶Q4(COQ4基因c.370G>A纯合变异；B：患儿之父COQ4基因c.370G>A杂合变异；C：患儿之母COQ4基因c.370G>A杂合变异；D：患儿之长兄COQ4基因c.370G野生型

后或认知障碍较为突出；21例(64%)患儿发病早期就有癫痫发作，以强直阵挛发作为主要临床表现；18例(55%)患儿出现不同程度的肌张力减退，尤其新生儿期明显。13例存在c.370 G>A位点变异的患儿均在新生儿期或婴儿期起病，且11例具有婴儿痉挛症、精神或生长发育障碍表现，仅2例患儿病程中未发现癫痫发作。20例(61%)患儿心脏彩色超声提示肥厚性心肌病(左心室为主)，部分患儿具有早期心力衰竭表现，但无心脏结构改变；26例有血清乳酸指标的患儿中有25例(96%)存在乳酸升高或乳酸酸中毒；28例(85%)患儿的颅脑影像学可见多种病变，如大脑或小脑发育不良、脑萎缩、基底节病变、胼胝体发育不良、额顶叶损伤等；5例患儿肌肉组织样本的辅酶Q10水平下降；多数患儿预后不佳，13例患儿在文献发表时仍存活但9例患儿对治疗效果反应较差，文献汇总病死20例，死亡年龄为4小时龄~3岁6月龄。

讨　　论

已发现有13个基因与辅酶Q10生物合成过程

中关键酶的编码相关，其中8种基因变异可导致辅酶Q10缺乏症。原发性辅酶Q10缺乏症的1~8型分别由COQ2、PDSS1、PDSS2、ADCK3、COQ9、COQ6、COQ4和COQ7基因变异导致，其主要有6种临床疾病：婴儿脑病、心肌病及肾功能衰竭，Leigh综合征，脑肌病、癫痫与共济失调，孤立的肌病，小脑综合征（小脑萎缩、共济失调），类固醇抵抗性肾病综合征[12]。

COQ4基因变异在临床表型、生化指标及颅脑影像学上有着明显的异质性，这可能与不同的COQ4基因变异位点或其他基因变异相关，这些基因变异甚至导致了原发性COQ10D7对辅酶Q10治疗反应的临床差异[1-3, 5-9]。与既往研究报道的多数病例不同的是[1, 5-9]，本例患儿未在新生儿期出现呼吸窘迫或呼吸功能不全，而以喂养困难、癫痫发作、肌张力减退为主要临床表现，头颅MRI显示右侧顶叶异常信号、白质髓鞘发育落后，未见大脑或小脑萎缩或基底神经节受影响。已有报道显示呼吸窘迫、癫痫发作、心肌肥厚以及乳酸酸中毒是COQ4基因变异导致的原发性COQ10D7常见的临床表现，尤其是新生儿期起病的病例，这提示上述临床表型可作为COQ4基因变异存在的重要线索。近年来部分研究表明辅酶Q10缺乏可导致线粒体病肾损伤或肾脏病，其临床表现为激素耐药性肾脏病，甚至将辅酶Q10缺乏相关蛋白尿及进展性肾病统称为"辅酶Q肾病"，其相关变异基因为COQ2、COQ6、COQ8和COQ9等[13]。与COQ2、COQ6、COQ7、COQ8和COQ9基因变异不同的是，COQ4基因变异的患儿均未发现肾脏损伤，这说明发病机制可能不同[14]。因此，肾脏是否损伤也是原发性COQ10D7一个有价值的诊断指标[1-3, 12]。

2012年，Salviati等[5]首次在1例具有智力障碍、脑肌病等临床特征的3岁男孩身上发现了染色体9q34.13缺失，而该缺失包含了COQ4基因，对其进行辅酶Q10缺乏的筛查发现，COQ4基因的表达比对照组低，继而导致了辅酶Q10含量、生物合成酶速率及线粒体呼吸链复合体（C）Ⅱ+Ⅲ活性明显低于对照组，该患儿口服补充辅酶Q10后其神经肌肉症状显著改善。2019年中国大陆首次报道了3例COQ4基因变异致原发性COQ10D7患儿[2-3]，1例3月龄男童来自北方地区的河南，临床上表现为喂养困难、癫痫、生长发育迟缓伴脑萎缩，基因检查显示COQ4基因复合错义变异：c.436T>A（p.F146I，父系来源）和c.211G>A（p.A71T，母系来源）[2]。2例来自南方福建地区同一家系，为COQ4纯合子变异c.370 G>A（p.G124S）导致辅酶Q10缺乏相关的Leigh综合征，其发病年龄为1~2月龄，表现为呼吸窘迫、癫痫、发育落后、肌张力障碍、乳酸酸中毒以及中脑、基底神经节病变[3]。Yu等[1]进一步研究表明体外培养自患者的成纤维细胞中辅酶Q10和线粒体呼吸链复合体（C）Ⅱ+Ⅲ活性水平明显低于未携带c.370 G>A者的，经过辅酶Q10处理后可使线粒体呼吸链复合体（C）Ⅱ+Ⅲ活性水平升高。同年，Lu等[3]在中国台湾和香港地区的11例原发性辅酶Q10缺乏症7型患儿中发现有10例COQ4基因存在c.370 G>A变异，认为COQ4基因c.370 G>A变异在中国南方的患者身上出现概率更高。此外，Yu等[1]利用ExAC数据库进行分析发现COQ4基因c.370 G>A变异只出现在东亚人群中，其等位基因频率为0.001 272（11/8 650，2018年8月8日）。同时对1 500名健康的中国成年人进行了该变异筛选，结果显示其等位基因频率为0.001。他们推断COQ4基因c.370 G>A纯合变异可能仅出现在东亚人群中。上述分析表明c.370G>A可能是中国南方地区原发性COQ10D7患儿的热点变异。

目前，原发性辅酶Q10缺乏症的治疗在于早期高剂量口服补充辅酶Q10［5~50 mg/(kg·d)］，可限制病情进展，逆转部分临床症状。然而，严重的神经和（或）肾损伤往往无法逆转。肥厚性心肌病、视网膜病和感音神经性听力损失的治疗是通常也难以完全可逆[12]。原发性COQ10D7患儿病死率高、预后欠佳且对辅酶Q10治疗存在较大的临床异质性，未来仍需对辅酶Q10补充治疗进行深入研究。临床上需对原发性COQ10D7随访监测，包括定期神经学评估，眼科评估，听力测试等。对有高危因素的亲属应进行症状前诊断，以便进行早期治疗并补充辅酶Q10。原发性辅酶Q10缺乏症作为一种常染色体隐性遗传，如果已知家庭中有致病变异，对高危亲属的携带者检测、对高危孕妇的产前检测以及植入前基因诊断都是可能的[12, 15-19]。葛丽丽等[2]报道了原发性COQ10D7一家系确诊后，患儿母亲进行再生育时成功通过胚胎植入前遗传学检测技术确保下一个后代不受影响。

综上所述，原发性辅酶Q10缺乏症罕见，COQ4基因变异分型主要导致神经肌肉系统损伤，但未发现累及肾脏。因此，对于临床上在新生儿期或婴儿期起病，有新生儿呼吸窘迫或呼吸功能不全、喂养困难、生长发育落后、癫痫、心肌肥大伴乳

酸升高等临床表型的患儿,建议行遗传学检测以进一步筛查该病可能,以期尽早诊治改善远期预后。

利益冲突 所有作者均声明不存在利益冲突

参 考 文 献

[1] Yu MH, Tsang MH, Lai S, et al. Primary coenzyme Q10 deficiency-7: expanded phenotypic spectrum and a founder mutation in southern Chinese[J]. NPJ Genom Med, 2019, 4:18. DOI: 10.1038/s41525-019-0091-x.

[2] 葛丽丽, 陈重芬, 刘磊, 等. COQ4 基因变异致原发性辅酶 Q10 缺乏症的临床特点和遗传学分析[J]. 中华内分泌代谢杂志, 2019, 35(12): 1014-1018. DOI: 10.3760/cma.j.issn.1000-6699.2019.12.004.

[3] Lu M, Zhou Y, Wang Z, et al. Clinical phenotype, in silico and biomedical analyses, and intervention for an East Asian population-specific c. 370G>A (p. G124S) COQ4 mutation in a Chinese family with CoQ10 deficiency-associated Leigh syndrome[J]. J Hum Genet, 2019, 64(4):297-304. DOI: 10.1038/s10038-019-0563-y.

[4] Green RC, Berg JS, Grody WW, et al. ACMG recommendations for reporting of incidental findings in clinical exome and genome sequencing[J]. Genet Med, 2013, 15(7):565-574. DOI: 10.1038/gim.2013.73.

[5] Salviati L, Trevisson E, Rodriguez Hernandez MA, et al. Haploinsufficiency of COQ4 causes coenzyme Q10 deficiency[J]. J Med Genet, 2012, 49(3): 187-191. DOI: 10.1136/jmedgenet-2011-100394.

[6] Brea-Calvo G, Haack TB, Karall D, et al. COQ4 mutations cause a broad spectrum of mitochondrial disorders associated with CoQ10 deficiency[J]. Am J Hum Genet, 2015, 96(2):309-317. DOI: 10.1016/j.ajhg.2014.12.023.

[7] Chung WK, Martin K, Jalas C, et al. Mutations in COQ4, an essential component of coenzyme Q biosynthesis, cause lethal neonatal mitochondrial encephalomyopathy[J]. J Med Genet, 2015, 52(9): 627-635. DOI: 10.1136/jmedgenet-2015-103140.

[8] Sondheimer N, Hewson S, Cameron JM, et al. Novel recessive mutations in COQ4 cause severe infantile cardiomyopathy and encephalopathy associated with CoQ10 deficiency[J]. Mol Genet Metab Rep, 2017, 12: 23-27. DOI: 10.1016/j.ymgmr.2017.05.001.

[9] Bosch AM, Kamsteeg EJ, Rodenburg RJ, et al. Coenzyme Q10 deficiency due to a COQ4 gene defect causes childhood-onset spinocerebellar ataxia and stroke-like episodes[J]. Mol Genet Metab Rep, 2018, 17: 19-21. DOI: 10.1016/j.ymgmr.2018.09.002.

[10] Romero-Moya D, Castaño J, Santos-Ocaña C, et al. Generation, genome edition and characterization of iPSC lines from a patient with coenzyme Q10 deficiency harboring a heterozygous mutation in COQ4 gene[J]. Stem Cell Res, 2017, 24: 144-147. DOI: 10.1016/j.scr.2016.09.007.

[11] Mak CC, Leung GK, Mok GT, et al. Exome sequencing for paediatric-onset diseases: impact of the extensive involvement of medical geneticists in the diagnostic odyssey[J]. NPJ Genom Med, 2018, 3: 19. DOI: 10.1038/s41525-018-0056-5.

[12] Salviati L, Trevisson E, Doimo M, et al. Primary coenzyme Q10 deficiency[M]//Adam MP, Ardinger HH, Pagon RA, et al. GeneReviews®. Seattle (WA): University of Washington, 2017.

[13] 曹琦, 李国民, 徐虹, 等. 辅酶 Q10 治疗 COQ6 基因突变致肾病一例并文献复习[J]. 中华儿科杂志, 2017, 55(2):135-138. DOI: 10.3760/cma.j.issn.0578-1310.2017.02.019.

[14] 徐可, 毛晓燕, 姚勇, 等. COQ2 基因变异致婴儿型肾病综合征一例临床分析并文献复习[J]. 中华儿科杂志, 2018, 56(9): 662-666. DOI: 10.3760/cma.j.issn.0578-1310.2018.09.006.

[15] Hernández-Camacho JD, Bernier M, López-Lluch G, et al. Coenzyme Q10 supplementation in aging and disease[J]. Front Physiol, 2018, 9: 44. DOI: 10.3389/fphys.2018.00044.

[16] Yubero D, Montero R, Santos-Ocaña C, et al. Molecular diagnosis of coenzyme Q10 deficiency: an update[J]. Expert Rev Mol Diagn, 2018, 18(6): 491-498. DOI: 10.1080/14737159.2018.1478290.

[17] Alcázar-Fabra M, Trevisson E, Brea-Calvo G. Clinical syndromes associated with coenzyme Q10 deficiency[J]. Essays Biochem, 2018, 62(3): 377-398. DOI: 10.1042/EBC20170107.

[18] Herebian D, López LC, Distelmaier F. Bypassing human CoQ10 deficiency[J]. Mol Genet Metab, 2018, 123(3): 289-291. DOI: 10.1016/j.ymgme.2017.12.008.

[19] Romero-Moya D, Santos-Ocaña C, Castaño J, et al. Genetic rescue of mitochondrial and skeletal muscle impairment in an induced pluripotent stem cells model of coenzyme Q10 deficiency[J]. Stem Cells, 2017, 35(7): 1687-1703. DOI: 10.1002/stem.2634.

·作者须知·

关于关键词的标引

我刊要求对论著类文章需标引 2～5 个关键词。请使用中国医学科学院医学信息研究所的《中文医学主题词表》(CmeSH)所列的词,或登录万方医学网:http://med.wanfangdata.com.cn/Mesh/Mesh.aspx 查找。如果查不到相应的关键词,处理办法有:(1)可选用直接相关的几个主题词进行组配。(2)可根据树状结构表选用最直接的上位主题词。(3)必要时,可采用习用的自由词并排列于最后。每组英文关键词第一个字母大写,各组词汇之间用“;”相隔。
